# Rottlerin, a natural polyphenol compound, inhibits upregulation of matrix metalloproteinase-9 and brain astrocytic migration by reducing PKC-δ-dependent ROS signal

**DOI:** 10.1186/s12974-020-01859-5

**Published:** 2020-06-06

**Authors:** Tsong-Hai Lee, Jiun-Liang Chen, Pei-Shan Liu, Ming-Ming Tsai, Su-Jane Wang, Hsi-Lung Hsieh

**Affiliations:** 1 Stroke Center and Stroke Section, Department of Neurology, College of Medicine, Chang Gung Memorial Hospital, Linkou Medical Center and Chang Gung University, Taoyuan, Taiwan; 2 Division of Chinese Internal Medicine, Center for Traditional Chinese Medicine, Chang Gung Memorial Hospital and School of Traditional Chinese Medicine, College of Medicine, Chang Gung University, Taoyuan, Taiwan; 3 Department of Microbiology, Soochow University, Taipei, Taiwan; 4 Department of Nursing, Division of Basic Medical Sciences, Research Center for Chinese Herbal Medicine, and Graduate Institute of Health Industry Technology, Chang Gung University of Science and Technology, 261 Wenhua 1st Road, Guishan, Taoyuan, Taiwan; 5 Department of General Surgery, Chang Gung Memorial Hospital, Chiayi, Taiwan; 6 School of Medicine, Fu Jen Catholic University, New Taipei City, Taiwan; 7 Department of Neurology, Chang Gung Memorial Hospital, Taoyuan, Taiwan

**Keywords:** Rottlerin, Matrix metalloproteinase-9, Protein kinase C-δ, Reactive oxygen species, Brain astrocytes, Neuroinflammation

## Abstract

**Background:**

Upregulation of matrix metalloproteinase-9 (MMP-9) has been indicated as one of the inflammatory biomarkers. In the central nervous system (CNS), the MMP-9 is induced by several proinflammatory mediators and participates in the CNS disorders, including inflammation and neurodegeneration. In addition, protein kinase Cs (PKCs) has been shown to be involved in regulation of various inflammatory factors like MMP-9 by several stimuli in many cell types. Several phytochemicals are believed to reduce the risk of several inflammatory disorders including the CNS diseases. The rottlerin, a principal phenolic compound of the Kamala plant *Mallotus philippinensi*s, has been shown to possess an array of medicinal properties, including anti-PKC-δ, antitumor, anti-oxidative, and anti-inflammatory activities.

**Methods:**

Herein, we used rat brain astrocytes (RBA) to demonstrate the signaling mechanisms of phorbol 12-myristate 13-acetate (PMA)-induced MMP-9 expression by zymographic, RT-PCR, subcellular isolation, Western blot, ROS detection, and promoter reporter analyses. Then, we evaluate the effects of rottlerin on PMA-induced MMP-9 expression in RBA and its influencing mechanism.

**Results:**

We first demonstrated that PMA stimulated activation of various types of PKC, including PKC-δ in RBA. Subsequently, PMA induced MMP-9 expression via PKCδ-mediated reactive oxygen species (ROS) generation, extracellular signal-regulated kinase 1/2 (ERK1/2) activation, and then induced c-Fos/AP-1 signaling pathway. Finally, upregulation of MMP-9 by PMA via the pathway may promote astrocytic migration, and the event could be attenuated by rottlerin.

**Conclusions:**

These data indicated that rottlerin may have anti-inflammatory activity by reducing these related pathways of PKC-δ-dependent ROS-mediated MMP-9 expression in brain astrocytes.

## Background

Natural medicinal plants are an important part of traditional medicine, an ancient complex therapy considered today as one of the most complete complementary medicine system. Recently, several natural products have been included into European Pharmacopoeia. The past decade has also witnessed an intense interest in natural herbal medicines in which phytochemical components can have long-term health promoting or medicinal qualities. Phytochemicals present in natural products such as herbs are believed to reduce the risk of several major diseases including cardiovascular diseases, cancers, and neurodegenerative disorders [1]. A likely mechanism of at least some of the activities is interacting with redox balance and prevention of oxidative stress. During the past two decades, hundreds of natural products, extracts, and isolated compounds have been screened for their antioxidant properties in vitro and in vivo. Consequently, some of natural products can be regarded as source of very efficient antioxidant compounds, and this activity could explain some of their therapeutic and preventive usefulness [2]. The rottlerin is a polyphenol natural product isolated from the Asian Kamala plant *Mallotus philippinensis* [3] and displays a complex spectrum of pharmacology and an array of medicinal properties. It has been used as a protein kinase C-δ (PKC-δ) inhibitor. Although there is extensive published documentation to support the use of rottlerin as a selective PKC-δ inhibitor, there has been some controversy in the literature over this claim [4]. It has been demonstrated to exhibit antitumor, autophagy, anti-proliferative, anti-metastasis, and anti-invasive properties [5–7]. In the central nervous system (CNS), previous report has indicated that several natural phenolic compounds like rottlerin may as potential neuroprotective agents to treat Parkinson’s disease [5]. However, the mechanisms of rottlerin in the CNS neuroprotective action remain unclear.

The astrocytes are one type of glial cells in the CNS, which have been proposed to exert a wide range of functions including participating in the immune and repairing responses to brain injury and diseases [8, 9]. Following injury to the human CNS, astrocytes become reactive and respond in stereotypical manner termed astrogliosis [10] which is characterized by astrocyte proliferation and functional changes in inflammatory diseases [11]. In brain, PKC and related kinases are activated during trauma, stroke, and neurogenic inflammation [12, 13], which may play a critical role in the initiation of the CNS inflammatory diseases. However, the effect of rottlerin on PKC-dependent MMP-9 expression is still unclear, although we have demonstrated that PKCs, PKC-δ especially, contribute to bradykinin-induced MMP-9 expression in brain astrocytes [14].

Matrix metalloproteinases (MMPs) are a large family of zinc-dependent endopeptidases which is a crucial molecule for the turnover of extracellular matrix (ECM) and pathophysiological processes [15]. In the CNS, MMPs, MMP-9 especially, has been demonstrated to participate in morphogenesis, wounding healing, and neurite outgrowth [16]. Several lines of evidence have showed that upregulation of MMP-9 may contribute to the pathogenic process of brain diseases by several brain injuries [17]. Moreover, several proinflammatory mediators such as cytokines and endotoxin have been shown to induce MMP-9 expression and activity in rat brain astrocytes [18, 19]. Our previous studies have showed that several proinflammatory mediators can induce MMP-9 expression and MMP-9-related functions in brain astrocytes [20]. These studies indicated that MMP-9 may play a critical role in brain inflammation and disorders, and this has aroused our interest to investigate the effects of natural products like rottlerin on MMP-9 expression in brain astrocytes. Here, we used the model of upregulation of MMP-9 by a PKC activator, phorbol 12-myristate 13-acetate (PMA), in brain astrocytes (RBA) to evaluate the effects of rottlerin on MMP-9 regulation and the relative events such as cell migration.

Reactive oxygen species (ROS) are produced by various enzymatic and chemical processes or directly inhaled, including O_2_•^−^, •OH, and hydrogen peroxide (H_2_O_2_). The ROS at low level have physiological roles as signaling molecules in various cellular and developmental processes [21, 22] and killing of invading microorganisms [23]. In contrast, recent report indicated that oxidative stress plays an important role in the progression of various diseases [23]. Moreover, ROS has been shown to interact with DNA, lipids, proteins, and carbohydrates that lead to cellular dysfunctions and inflammatory responses [22, 24]. Under pathological conditions, many proinflammatory mediators induce expression of several inflammatory genes during brain injury via increasing ROS production [20, 22, 25]. Moreover, increasing evidence attributes the neurodegenerative diseases such as Alzheimer’s disease (AD) to oxidative stress (generation of free radicals) that leads to brain inflammation during CNS pathogenesis [22, 25, 26]. Moreover, ROS also exert as a signaling factor mediated microglial activation induced by several proinflammatory mediators [27]. The effects of PKCs associated with ROS generation have been reported in several organ diseases [28, 29]. Our previous reports indicated that ROS is critical for upregulation of MMP-9 responses in rat brain astrocytes [30, 31].

Based on these backgrounds and our previous studies in the brain inflammatory responses by MMP-9 induction [20], the experiments were performed to evaluate the effects and molecular mechanisms of rottlerin on PMA-induced MMP-9 expression in brain astrocytes (RBA). In the study, we found that the rottlerin reduced PMA-induced MMP-9 expression and astrocytic migration. Moreover, PMA-stimulated phosphorylation of protein kinases (e.g., PKC-δ, ROS, and ERK1/2) also been inhibited by pretreatment of rottlerin. Furthermore, the rottlerin decreased PKC-δ-mediated Nox/ROS/ERK-dependent activation of c-Fos/AP-1 pathway in RBA cells. These results suggested that the rottlerin may be has neuroprotective effects by anti-oxidative and anti-inflammatory action in the CNS.

## Methods

### Materials

Dulbecco’s modified Eagle’s medium (DMEM)/F-12 medium, fetal bovine serum (FBS), and TRIzol were from Invitrogen (Carlsbad, CA). Hybond C membrane and enhanced chemiluminescence (ECL) Western blot detection system were from GE Healthcare Biosciences (Buckinghamshire, UK). PKC isotypes (PKC-α、PKC-β、PKC-γ、PKC-ε、PKC-δ) and PhosphoPlus ERK1/2 (Thr^202^/Tyr^204^) antibodies were from Cell Signaling (Danver, MA). Anti-glyceraldehyde-3-phosphate dehydrogenase (GAPDH) antibody was from Biogenesis (Boumemouth, UK). Rottlerin, apocynin, PD98059, tanshinone IIA (TSIIA), and MMP2/9 inhibitor (2/9i) were from Biomol (Plymouth Meeting, PA). Bicinchoninic acid (BCA) protein assay reagent was from Pierce (Rockford, IL). Phorbol 12-myristate 13-acetate (PMA), n-acetyl-cysteine (NAC), enzymes, and other chemicals were from Sigma (St. Louis, MO).

### Cell cultures and treatments

The rat brain astrocytic cell line (RBA, CTX TNA2) was purchased from BCRC (Hsinchu, Taiwan) and used throughout this study. Cells were plated onto 12-well culture plates and made quiescent at confluence by incubation in serum-free DMEM/F-12 for 24 h, and then incubated with PMA (0.01 ~ 10 μM) at 37 °C for the indicated time intervals. When the inhibitors were used, cells were pretreated with the inhibitor for 1 h before exposure to PMA (1 μM). Treatment of RBA with these inhibitors alone had no significant effect on cell viability determined by an XTT assay (data not shown).

### MMP gelatin zymography

Growth-arrested cells were incubated with PMA for the indicated time intervals. After treatment, the cultured media were collected and analyzed by gelatin zymography [32]. Gelatinolytic activity was manifested as horizontal white bands on a blue background. Because cleaved MMPs were not reliably detectable, only pro-form zymogens were quantified.

### Total RNA extraction and reverse transcription-PCR analysis

Total RNA was extracted from RBA cells [33]. The cDNA obtained from 0.5 μg total RNA was used as a template for PCR amplification. Oligonucleotide primers were designed on the basis of Genbank entries for rat MMP-9, c-Fos, and β-actin. The primers were:

*MMP-9*: 5′-AGTTTGGTGTCGCGGAGCAC-3′ (sense)

5′-TACATGAGCGCTTCCGGCAC-3′ (antisense)

*c-Fos*: 5′-AGACGAAGGAAGACGTGTAAGCACTGCAGCT-3′ (sense)

5′- AAGGAGAATCCGAAGGGAAAGGAATAAGATG-3′ (antisense)

*β-actin*: 5′-GAACCCTAAGGCCAACCGTG-3′ (sense)

5′-TGGCATAGAGGTCTTTACGG-3′ (anti-sense)

The amplification was performed in 30 cycles at 55 °C, 30 s; 72 °C, 1 min; 94 °C, 30 s. PCR fragments were analyzed on 2% agarose 1X TAE gel containing ethidium bromide, and their size was compared with a molecular weight markers. Amplification of β-actin, a relatively invariant internal reference RNA, was performed in parallel, and cDNA amounts were standardized to equivalent β-actin mRNA levels. The image densitometry analysis was quantified by an UN-SCAN-IT gel 6.1 software (Orem, UT).

### Preparation of subcellular fractions and detection of PKC isoforms translocation

RBA cells were seeded in a 10-cm dish. After reaching 90% confluence, the cells were starved for 24 h in serum-free medium and treated with PMA (1 μM) for the indicated times. The cells were washed once with ice-cold phosphate-buffered saline (PBS). Two hundred microliter of homogenization buffer A (20 mM Tris-HCl, pH 8.0, 10 mM EGTA, 2 mM EDTA, 2 mM dithiothreitol, 1 mM phenylmethylsulfonyl fluoride, 25 mM aprotinin, 10 mM leupeptin) was added to each dish, and the cells were scraped into a 1.5-mL tube with a rubber policeman. The cytosolic and membrane fractions were prepared by centrifugation as described previously [34].

### Preparation of cell extracts and western blot analysis

Growth-arrested cells were incubated with PMA at 37 °C for the indicated time intervals. The cells were washed with ice-cold PBS, scraped, and collected by centrifugation at 45,000 × *g* for 1 h at 4 °C to yield the whole cell extract, as previously described [14]. Samples were analyzed by Western blot, transferred to nitrocellulose membrane, and then incubated overnight using an anti-PKC isotypes (e.g., PKC-α, PKC-β, PKC-γ, PKC-ε, and PKC-δ), anti-phospho-ERK1/2, ERK2, or GAPDH antibody. Membranes were washed four times with TTBS for 5 min each, incubated with a 1:2000 dilution of anti-rabbit horseradish peroxidase antibody for 1 h. The immunoreactive bands were detected by ECL reagents and captured by a UVP BioSpectrum 500 Imaging System (Upland, CA). The image densitometry analysis was quantified by an UN-SCAN-IT gel 6.1 software (Orem, UT).

### Measurement of intracellular ROS generation

The peroxide-sensitive fluorescent probe 2′,7′-dichlorofluorescein diacetate (DCF-DA) was used to assess the generation of intracellular ROS [35] with minor modifications. RBA cells on monolayers were incubated with 5 μM DCF-DA in RPMI-1640 at 37 °C for 45 min. The supernatant was removed and replaced with fresh RPMI-1640 medium before exposure to PMA (1 μM). Relative fluorescence intensity was recorded at the indicated time by using a fluorescent plate reader (Thermo, Appliskan) at an excitation wavelength of 485 nm, and emission was measured at a wavelength of 530 nm. The fluorescent images were also obtained by using fluorescence microscopy (Axiovert 200 M; Zeiss).

### MMP-9 promoter-luciferase reporter gene assay

The upstream region (− 1280 to + 19) of the rat MMP-9 promoter was cloned to the pGL3-basic vector containing the luciferase reporter system [14]. All plasmids were prepared by using QIAGEN plasmid DNA preparation kits. These constructs were transfected into RBA cells by using a Lipofectamine reagent according to the instructions of the manufacture. The transfection efficiency (~ 60%) was determined by transfection with enhanced GFP. After incubation with PMA, cells were collected and disrupted by sonication in lysis buffer (25 mM Tris, pH 7.8, 2 mM EDTA, 1% Triton X-100, and 10% glycerol). After centrifugation, aliquots of the supernatants were tested for promoter activity using a luciferase assay system (Promega, Madison, WI). Firefly luciferase activities were standardized for β-galactosidase activity.

### Cell migration assay

RBA cells were cultured to confluence in 6-well plates and starved with serum-free DMEM/F-12 medium for 24 h. The monolayer cells were scratched manually with a blade, and the detached cells were removed with PBS. Serum-free DMEM/F-12 medium with or without PMA (1 μM) was added to each dish as indicated after pretreatment of inhibitors for 1 h, containing a DNA synthesis inhibitor hydroxyurea (10 μM) during the period of experiment. Images were observed and taken at 0 and 24 h with a digital camera and a microscope (Olympus, Japan). These resulting four phase images for each point were averaged and then normalized based on the initial image at 0 h. The normalized values were averaged for each experimental condition. The data presented are generated from three separate assays.

### Statistical analysis of data

All data were estimated using GraphPad Prism Program (GraphPad, San Diego, CA). Quantitative data were analyzed by one-way ANOVA followed by Tukey’s honestly significant difference tests between individual groups. Data were expressed as mean ± SEM. A value of *P* < 0.05 was considered significant.

## Results

### Phorbol 12-myristate 13-acetate (PMA) induces MMP-9 expression in brain astrocytes

**Fig. 1:**
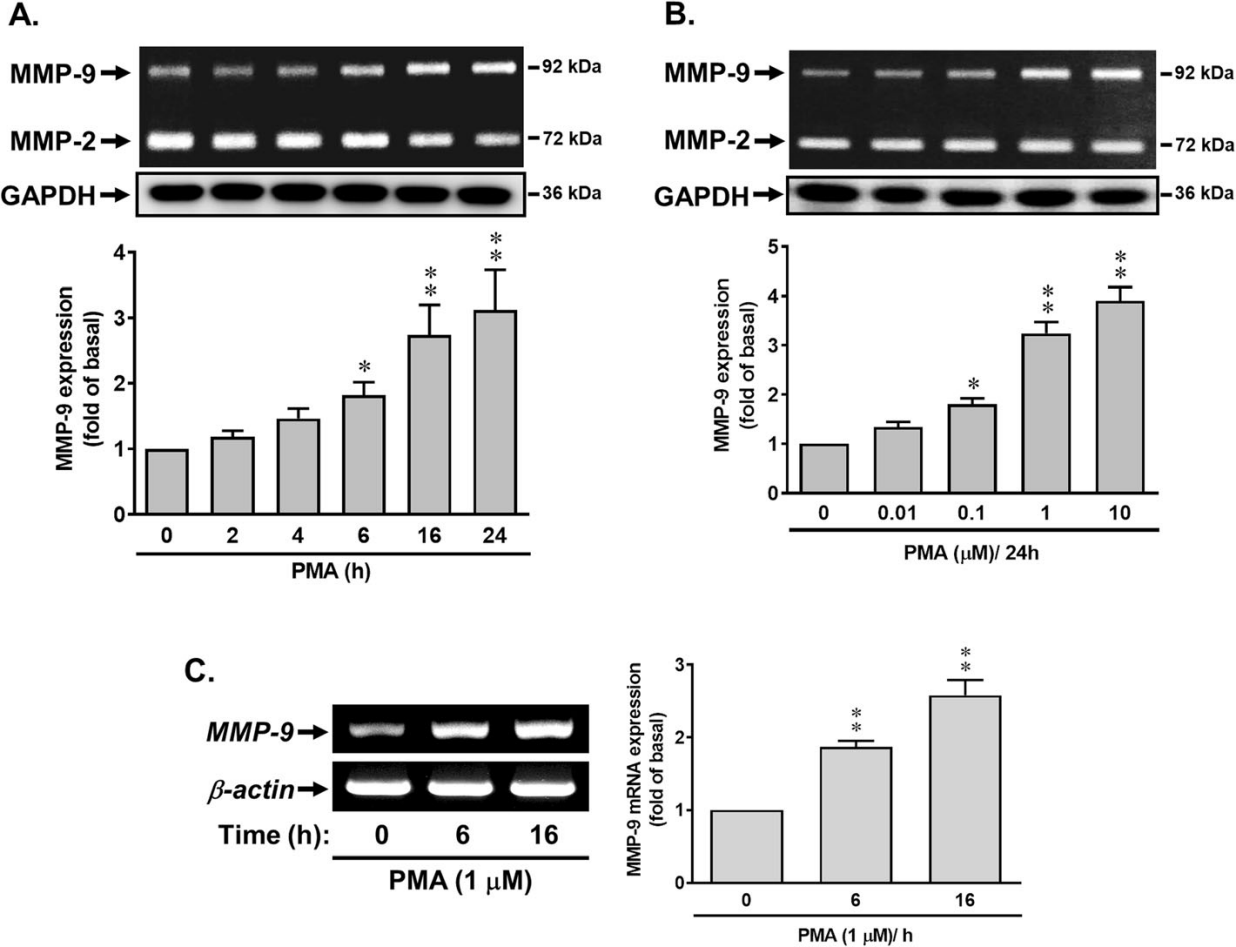
Phorbol 12-myristate 13-acetate (PMA), a PKC activator, induces MMP-9 expression, but not MMP-2, in brain astrocytes (RBA). **a** Time dependence of PMA increase of MMP-9 expression. RBA cells were treated with 1 μM PMA for the indicated time intervals. **b** Concentration dependence of PMA-induced MMP-9 expression. Cells were treated with various concentrations of PMA (0.01, 0.1, 1, and 10 μM) for 24 h. **c** Time dependence of PMA-induced MMP 9 mRNA expression. Cells were treated with 1 μM PMA for the indicated times. The conditioned media, cell lysates, and total RNA were collected and analyzed by gelatin zymography (MMP2/9), Western blot (GAPDH, as an internal control), and RT-PCR (MMP-9 and β-actin) as described under the “Methods” section. The intensity of zymographic (**a**, **b**) and PCR product (**c**) bands was quantitated by scanning densitometry and expressed as fold of untreated control. Data are expressed as the mean ± SEM (*N* = 3). *P < 0.05; **P < 0.01, as compared with the respective values of untreated control. The image represents one of three individual experiments

The PKCs are crucial for regulation of MMP-9 in brain inflammatory diseases [14]. First, we investigate whether the PKC activator, phorbol 12-myristate 13-acetate (PMA), can upregulate MMP-9 expression in rat brain astrocytes (RBA), cells were treated with PMA (1 μM) for the indicated time intervals, and then the conditioned media were collected and analyzed. As shown in Fig. 1a, treatment with PMA (1 μM) significantly induced MMP-9 expression in a time-dependent manner determined by gelatin zymography. The expression of a housekeeping protein GAPDH, as an internal control, was not changed. There was a significant increase between 6 and 24 h. A maximal increase was achieved at 24 h during the period of observation. Moreover, the RBA cells were incubated with different concentrations of PMA (0.01, 0.1, 1, and 10 μM) for 24 h. In addition, RBA cells were incubated with various concentrations of PMA (0.01, 0.1, 1, and 10 μM) for 24 h, and the MMP-9 was determined by gelatin zymography (Fig. 1b). The induction response of PMA was in a concentration-dependent increase, and there was a significant increase from 100 nM of PMA. A maximal induction of PMA was obtained at the concentration of 10 μM. To further examine whether the increase in MMP-9 expression by PMA resulted from the increase of MMP-9 mRNA expression, the RT-PCR analysis was performed. As shown in Fig. 1c, the levels of mRNA for MMP-9 in RBA cells were determined by RT-PCR. PMA time dependently induced the mRNA expression of MMP-9 in the cells, whereas the expression of β-actin mRNA, a housekeeping gene product used as an internal control, was not changed. These data revealed that PMA induced the expression of MMP-9 by increasing mRNA level and suggested that PKCs might play an upregulatory role in PMA-induced MMP-9 expression in RBA cells.

### Effects of rottlerin, a natural polyphenol compound, on PMA-induced MMP-9 expression

**Fig. 2:**
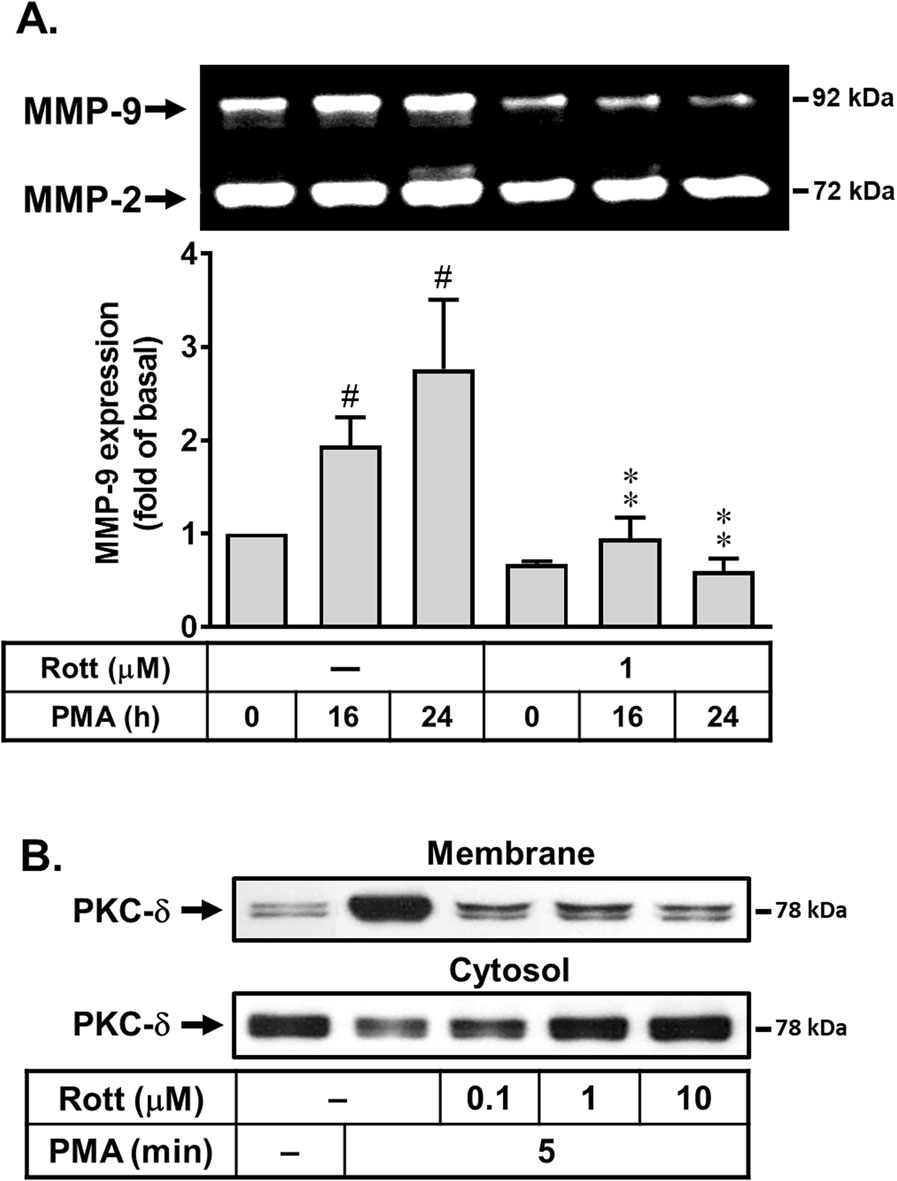
Effects of rottlerin, a natural polyphenol compound, on PMA-induced MMP-9 expression in RBA cells. **a** Time dependence of rottlerin inhibited PMA-induced MMP-9 expression. Cells were pretreated with or without rottlerin (Rott, 1 μM) and then incubated with PMA (1 μM) for the indicated time intervals. **b** Rottlerin concentration dependently inhibited PMA-stimulated PKC-δ translocation. Cells were pretreated with rottlerin (1 μM) for 1 h and then incubated with PMA (1 μM) for 5 min. The conditioned media (**a**) and subcellular fractions (**b**) were collected and assayed for MMP-9 expression by gelatin zymography (**a**) and Western blot with various PKC antibodies as described under the “Materials and Methods” section (**b**). Data are expressed as the mean ± SEM (*N* = 3). ^#^*P* < 0.01, as compared with the respective values of untreated control. *P < 0.05; **P < 0.01, as compared with the respective values of cells stimulated with PMA only. The image represents one of three individual experiments

Our previous data have shown that upregulation of MMP-9 requires PKC-δ-mediated manner [14]. To determine the role of PKC-δ in PMA-induced upregulation of MMP-9, a selective PKC-δ inhibitor rottlerin (a natural polyphenol compound) was used. The cells were pretreated with rottlerin (1 μM) for 1 h and then incubated with PMA for the indicated times. As shown in Fig. 2a, pretreatment with rottlerin caused a significant inhibition on PMA-induced MMP-9 expression revealed by gelatin zymography, suggesting that PKC (PKC-δ especially) may play a potential role in PMA-induced MMP-9 expression in RBA cells. Moreover, we also found that PMA stimulated several PKC isoform translocation, including PKC-α, PKC-βI, PKC-γ, PKC-ε, and PKC-δ (data not shown). Next, to determine the effect of rottlerin on PMA-stimulated PKC-δ translocation, the cells were pretreated with various concentration of rottlerin (0.1, 1, and 10 μM), and then the PKC-δ translocation was analyzed. The results showed that rottlerin concentration dependently inhibited PMA-stimulated membrane translocation of PKC-δ analyzed by Western blot (Fig. 2b). These results suggested that rottlerin may block PMA-induced MMP-9 expression via blocking PKC-δ activation in RBA cells.

### Role of rottlerin in PMA-induced MMP-9 expression via Nox-mediated ROS production

**Fig. 3:**
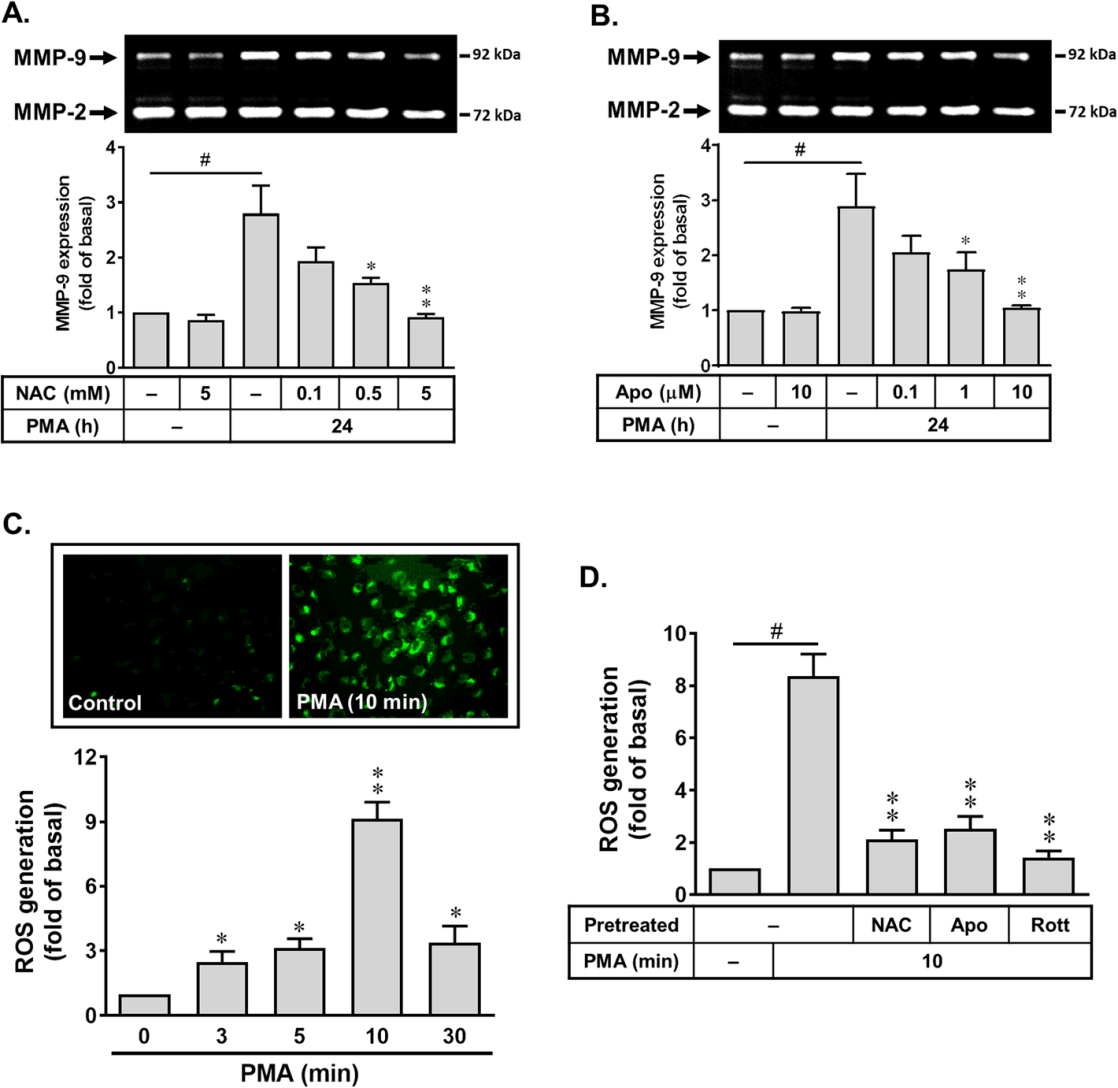
Roles of rottlerin in PMA-induced MMP-9 expression via Nox-mediated ROS production. **a**, **b** The Nox/ROS system inhibitor concentration dependently blocked PMA-induced MMP-9 expression, and cells were pretreated with **a** NAC (0.1, 1, and 5 mM) or (**b**) apocynin (Apo, 0.1, 1, 10 μM) and then incubated with PMA (1 μM) for 24 h. **c**, **d** PMA stimulates ROS generation. Cells were incubated with the DCF-DA (5 μM) for 45 min, followed by stimulation with PMA (1 μM) for the indicated times (**c**), or pretreatment with NAC (5 mM), Apo (10 μM), and Rott (1 μM) for 1 h and then stimulation with PMA (1 μM) for 10 min (**d**). The conditioned media were collected and assayed for MMP-9 expression by gelatin zymography (**a**, **b**). The fluorescence intensity (ROS generation) and images of cells (**c**, **d**) were determined as described in the “Methods” sectopn. Data are expressed as the mean ± SEM (*N* = 3). ^#^*P* < 0.01, as compared with the respective values of untreated control. *P < 0.05; **P < 0.01, as compared with the respective values of untreated cells (**c**) or cells stimulated with PMA only (**a**, **b**, **d**). The image represents one of three individual experiments

Recent report has indicated that ROS may contribute to MMP expression in various cell types [36]. The NADPH oxidase (Nox) is considered to be a major source of ROS in many physiological and pathological processes [23, 37]. Previous studies have demonstrated that Nox-derived ROS signaling cascade is involved in upregulation of MMP-9 by BK in astrocytes [30]. Thus, to determine whether rottlerin-reduced PMA-induced MMP-9 is due to decreasing Nox-dependent ROS production, the ROS scavenger n-acetylcysteine (NAC) and a Nox activity inhibitor apocynin were used. As shown in Fig. 3a, pretreatment with NAC (0.1, 0.5, or 5 mM) markedly inhibited PMA-induced MMP-9 expression in a concentration-dependent manner. Moreover, pretreatment with apocynin (Apo, 0.1, 1, or 10 μM) also blocked PMA-induced MMP-9 expression in a concentration-dependent manner (Fig. 3b). The data suggested that Nox-derived ROS generation may play a potential role in PMA-induced MMP-9 expression in RBA cells. To explore whether PMA induces ROS generation, the cells were loaded with DCF-DA (a ROS probe) and then stimulated with PMA (1 μM) for the indicated time intervals. As shown in Fig. 3c, PMA stimulated a time dependently ROS production with a maximal response within 10 min and sustained over 30 min. The results were further supported by the data of fluorescence images obtained using a fluorescent microscopy (Fig. 3c, internal panel). The image data showed that PMA stimulated ROS generation in RBA cells. Pretreatment of cells with NAC (5 mM) and Apo (1 μM) markedly attenuated PMA-stimulated ROS generation (Fig. 3d). Additionally, to examine the effects of rottlerin on PMA-stimulated ROS generation, the cells were pretreated with rottlerin (Rott, 1 μM) for 1 h and then incubated with PMA for 10 min. As shown in Fig. 3d, PMA-stimulated ROS generation was markedly attenuated by pretreatment with Rott, suggesting that upregulation of MMP-9 by PMA is mediated through a PKC-δ-dependent Nox-derived ROS production in RBA cells. Herein, we also demonstrated that rottlerin may be possessed of anti-oxidative effect in the event.

### Rottlerin attenuates upregulation of MMP-9 by PMA through blocking the Nox/ROS-mediated ERK1/2 activation in RBA cells

**Fig. 4:**
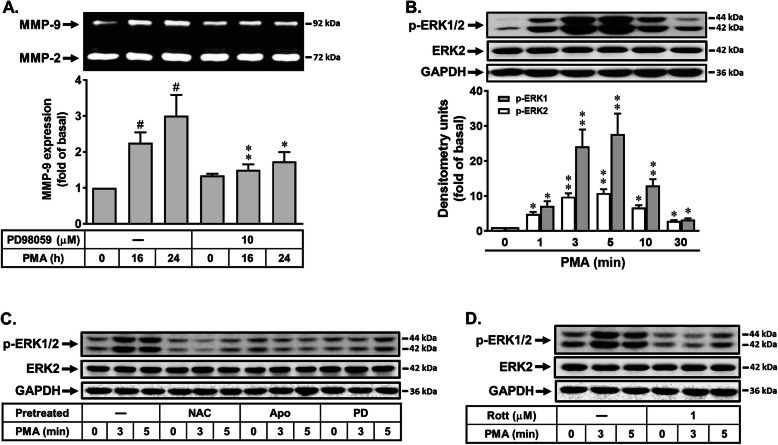
Rottlerin attenuates upregulation of MMP-9 by PMA through blocking the Nox/ROS-mediated ERK1/2 activation in RBA cells. **a** Cells were pretreated with PD98059 (10 μM) and then incubated with PMA (1 μM) for the indicated time intervals. **b** Cells were treated with PMA (1 μM) for the indicated times. **c**, **d** Cells were pretreated with NAC (5 mM), Apo (10 μM), PD98059 (PD, 10 μM), and Rott (1 μM) for 1 h and then stimulation with PMA (1 μM) for the indicated times. The conditioned media were collected and assayed for MMP-9 expression by gelatin zymography (**a**). The cell lysates were collected and analyzed phosphorylation of ERK1/2 (p-ERK1/2), total ERK2, and GAPDH by Western blot (**b**–**d**) as described in the “Methods” section. Data are expressed as the mean ± SEM (*N* = 3). ^#^*P* < 0.01, as compared with the respective values of untreated control. *P < 0.05; **P < 0.01, as compared with the respective values of untreated cells (**a**) or cells stimulated with PMA only (**b**). The image represents one of three individual experiments

Activation of MAPKs by various stimuli could modulate cellular functions of brain cells [32, 38]. Moreover, previous reports have pointed out that ERK1/2 is critical for the regulation of MMP-9 expression in brain astrocytes [14]. Thus, to determine whether ERK1/2 also participated in PMA-induced MMP-9 expression, cells were pretreated with or without PD98059 (10 μM) for 1 h and then incubated with PMA for the indicated time intervals. As shown in Fig. 4a, pretreatment with PD98059 attenuated PMA-induced MMP-9 expression, suggesting that ERK1/2 may be involved in PMA-induced MMP-9 expression. We further demonstrated that PMA stimulated time dependently ERK1/2 phosphorylation with a maximal response within 5 min and sustained over 30 min by Western blot (Fig. 4b). These results suggested that PMA-induced MMP-9 expression is mediated through ERK1/2 pathway in RBA cells. Next, to determine whether PMA-stimulated ERK1/2 phosphorylation is mediated through Nox/ROS-dependent pathway, cells were pretreated with NAC, Apo, or PD98059 (PD) and then incubated with PMA (1 μM) for the indicated time intervals. The results showed that pretreatment with NAC (5 mM), Apo (1 μM), or PD (10 μM) significantly attenuated PMA-stimulated ERK1/2 phosphorylation during the period of observation (Fig. 4c), suggesting that PMA stimulated Nox/ROS-dependent phosphorylation of ERK1/2 in these cells. Moreover, we further evaluated the effect of rottlerin in phosphorylation of ERK1/2 by PMA. As shown in Fig. 4d, pretreatment with rottlerin (Rott, 1 μM) markedly attenuated PMA-stimulated ERK1/2 phosphorylation, suggesting that activation of ERK1/2 may be involved in PMA-induced MMP-9 expression through PKC-δ-dependent manner. These results demonstrated that rottlerin blocked PMA-induced MMP-9 expression is mediated through inhibiting Nox/ROS-dependent ERK1/2 activation in RBA cells.

### Effects of rottlerin in PMA-stimulated activation of transcription factors such as AP-1

**Fig. 5:**
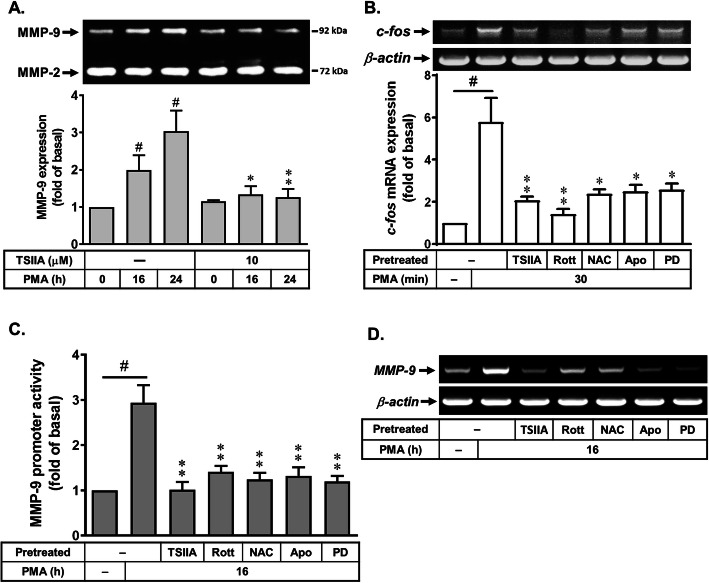
Effects of rottlerin in PMA-stimulated activation of transcription factors such as AP-1. **a** Cells were pretreated with tanshinone IIA (TSIIA, 10 μM) and then incubated with PMA (1 μM) for the indicated time intervals. The conditioned media were collected and assayed for MMP-9 expression by gelatin zymography. **b**, **d** Cells were pretreated with TSIIA (10 μM), NAC (5 mM), Apo (10 μM), PD98059 (PD, 10 μM), and Rott (1 μM) for 1 h and then stimulation with PMA (1 μM) for 30 min (**b**) or 16 h (**d**). The total RNA was collected and analyzed *c-fos* mRNA (30 min) and *MMP-9* mRNA (16 h) expression by RT-PCR analysis as described in the “Methods” section. **c** Cells were transiently transfected with pGL-MMP9-Luc and pGal for 24 h, pretreated with TSIIA (10 μM), NAC (5 mM), Apo (10 μM), PD98059 (PD, 10 μM), and Rott (1 μM) for 1 h and then stimulation with PMA (1 μM) for 16 h. After stimulation, luciferase activity of MMP-9-promoter construct was measured as relative promoter activity to that of β-galactosidase. Data are expressed as the mean ± SEM (*N* = 3). ^#^*P* < 0.01, as compared with the respective values of untreated control. *P < 0.05; **P < 0.01, as compared with the respective values of cells stimulated with PMA only. The image represents one of three individual experiments

The AP-1-dependent pathways have been demonstrated to involve in MMP-9 expression in various cell types [20]. We first determine whether PMA-induced MMP-9 expression is mediated through activation of AP-1 (e.g., c-Fos). As shown in Fig. 5a, pretreatment with an AP-1 inhibitor tanshinone IIA (TSIIA, 10 μM) significantly inhibited PMA-induced MMP-9 expression, suggesting that the transcription factor AP-1 may be involved in PMA-induced MMP-9 expression. Furthermore, the results of RT-PCR analysis showed that PMA can induce c-Fos, a subunit of AP-1, mRNA expression (~ 6-fold), pretreatment with TSIIA (10 μM), NAC (5 mM), Apo (1 μM), or PD (10 μM) attenuated PMA-stimulated c-Fos/AP-1 mRNA expression (Fig. 5b). These results demonstrated that PMA induced MMP-9 expression via Nox/ROS/ERK1/2-mediated activation of c-Fos/AP-1 cascade in RBA cells. Next, to evaluate the effects of rottlerin on PMA-induced c-Fos/AP-1 gene expression, the results were obtained by RT-PCR analysis. The results showed that pretreatment of RBA with rottlerin (Rott, 1 μM) significantly reduced PMA-stimulated c-Fos/AP-1 gene expression (Fig. 5b). Moreover, previous studies indicated that MMP-9 promoter region contains AP-1 binding sites [39]. Hence, we next examined whether rottlerin also affects PMA-induced MMP-9 promoter activity by blocking AP-1 activation, a rat MMP-9 promoter reporter constructs (pGL-MMP-9-Luc) was used [30, 39]. The data showed that pretreatment with TSIIA, NAC, Apo, PD, or rottlerin significantly attenuated PMA-increased MMP-9 promoter activity (Fig. 5c). We further demonstrated that PMA-induced MMP-9 mRNA expression was also mediated through this pathway determined by RT-PCR analysis (Fig. 5d). These results confirmed that PMA-induced MMP-9 mRNA expression is mediated through Nox/ROS/ERK-mediated upregulation of c-Fos/AP-1 linking to the MMP-9 promoter activity in RBA cells. In addition, rottlerin may play a suppressor in PMA-induced MMP-9 expression via reducing c-Fos/AP-1-mediated MMP-9 transcription activity in these cells.

### Rottlerin affects the PMA-induced MMP-9-mediated astrocytic migration

**Fig. 6:**
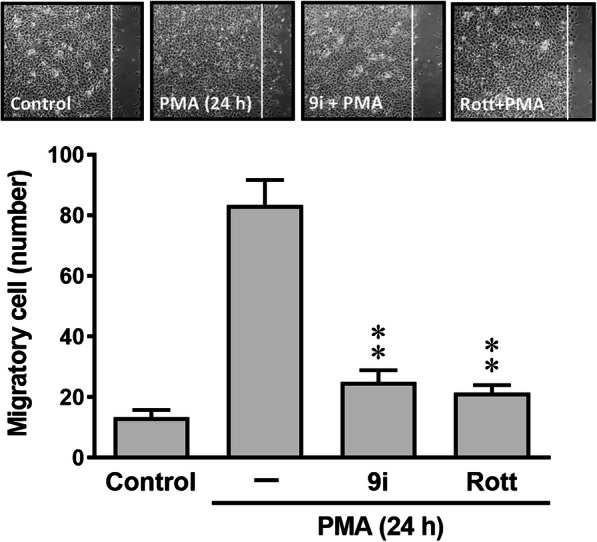
Rottlerin affects the PMA-induced MMP-9-mediated astrocytic migration. Cells were plated on 6-well culture plates, grew to confluence, and starved with serum-free medium for 24 h. Cells were pretreated with MMP-9 inhibitor (2/9i, 1 μM) or Rott (1 μM) for 1 h, and the monolayer cells were manually scratched with a blue tip as described in the “Methods” section and then incubated with PMA (1 μM) for 24 h. Phase contrast images of cells were taken at 24 h (upper panel), and the number of cell migration was counted (lower panel) as described in the “Methods” section. Data are expressed as mean ± SEM of three independent experiments (*N* = 3). ^#^*P* < 0.01, as compared with the respective values of untreated control. **P < 0.01, as compared with the values of cells stimulated with PMA only

The MMP-9 has been reported to be elevated in various brain injuries and participates in the pathogenesis of several CNS disorders. Moreover, upregulation of MMP-9 has been shown to involve in brain inflammation and cell migration [14, 40]. Therefore, we further investigated the effects of rottlerin on PMA-induced MMP-9-mediated cell functional changes such as cell migration. First, the images of RBA cell migration were observed and taken at 24 h after treatment of PMA (1 μM). Pretreatment with MMP-9 inhibitor (9i, 1 μM) significantly blocked PMA-induced RBA cell migration (Fig. 6, upper panel), demonstrating that the PMA-induced MMP-9 expression led to RBA cell migration. Next, we evaluated the effects of rottlerin on the cell migration response, and cells were pretreated with rottlerin (Rott, 1 μM) and then incubated with PMA for 24 h. The data showed that PMA-induced RBA cell migration was inhibited by pretreatment of rottlerin (Rott). The number of migratory RBA cells was counted, and the statistical data are presented in Fig. 6 (lower panel). The results demonstrated that rottlerin repressed PMA-induced cell migration via reducing MMP-9 expression in brain astrocytes.

## Discussion

MMPs contribute to a wide range of biological activities in different tissues, including several CNS diseases, such as stroke, Alzheimer’s disease, and malignant glioma [17]. Among MMPs, MMP-9 expression and activation play a critical role in tissue remodeling in the pathogenesis of brain diseases [17]. Reduction of MMP activity by pharmacological inhibitors or gene knock-out strategies protects the brain from BBB disruption, cell death, and advanced neuroinflammation [41]. These studies suggest that upregulation of MMP-9 by pro-inflammatory factors may be a great effect upon brain injury, inflammation, and neurodegeneration. Therefore, the inhibition of MMP-9-mediated inflammatory pathways may provide therapeutic strategies to brain inflammation and neurodegenerative diseases. Moreover, BK and related peptides are simultaneously produced and released following brain injury [20]. Our previous data have demonstrated that PKCs, PKC-δ especially, contribute to upregulation of MMP-9 in astrocytes which may change astrocytic functions such as cell motility and neuroinflammation [30, 39]. These findings of PKC-mediated MMP-9 expression in RBA imply that PKCs may play an important role in brain injury, astroglioma, or CNS diseases. Pharmacological and knockout-mouse approaches suggest that targeting MMP-9 and their upstream signaling pathways should yield useful therapeutic targets for brain injury, tumor, and neuroinflammation. Herein, we evaluate whether the natural product rottlerin possess anti-oxidative and anti-inflammatory effects on PMA-induced MMP-9 expression in brain astrocytes and its inhibitory mechanism. The results suggest that in brain astrocytes, the rottlerin-reduced PMA-induced MMP-9-dependent astrocytic migration is mediated through inhibition of the PKC-δ-activated Nox/ROS/ERK signal leading to induction of c-Fos/AP-1 pathway.

Here, we first found that a natural compound rottlerin can inhibit PMA-induced MMP-9 gene expression in RBA cells (Fig. 2). This result is the first finding that rottlerin can suppress MMP-9 upregulation by PMA in brain astrocytes. Next, many reports and our previous data have indicated that PKCs may contribute to various stimuli-induced MMP-9 expression in brain astrocytes [14, 30]. Moreover, several reports also demonstrate that PKC-δ is crucial for MMP-9 expression [14]. Thus, we investigated whether the inhibition of rottlerin is mediated through blocking the activation of PKC signals, PKC-δ especially, by PMA in brain astrocytes. The results showed that PMA stimulated PKC activation (translocation), including PKC-δ, which were attenuated by pretreatment with rottlerin in RBA cells (Fig. 2). These data demonstrated that rottlerin may inhibit PMA-induced MMP-9 expression via reducing PKC (i.e., PKC-δ)-mediated pathways in RBA cells.

Redox imbalance plays a causative role in numerous pathologies of degenerative diseases [26]. ROS concentration dependently exerts a key role in the normal physiological functions and the inflammatory responses [24]. In the brain, ROS also extend to the control of vascular tone which is tightly modulated by metabolic activity within neurons [25]. Moreover, increasing ROS generation by diverse stimuli can regulate the expression of inflammatory genes in pathogenesis of brain disorders [42]. Recently, the cellular damage in neurodegenerative disorders such as Alzheimer’s disease (AD) is attributed to oxidative stress in brain inflammatory disorders [22, 26]. In astrocytes, our recent data have demonstrated that in both in vitro and in vivo studies, the ROS-dependent pathways contribute to upregulation of MMP-9 in brain astrocytes. Moreover, we found that upregulation of MMP-9 by BK is mediated through Nox-mediated ROS generation [30]. In the study, we further demonstrated that rottlerin may have an anti-oxidative activity (Fig. 3). Herein, we are the first group to establish that rottlerin reduce Nox/ROS signal induced by PMA in brain astrocytes. The finding is consistent with previous study indicated that PKC-δ phosphorylation is an upstream event of GSK3 inactivation-mediated ROS generation in TGF-β1-induced senescence [43]. It is consistent with previous report indicated that rottlerin induces cyclooxygenase-2 upregulation through reactive oxygen species-independent pathway in HEI-OC1 cells [44].

The MAPK regulation has been reported to act as an important inflammatory event through activation of MAPK cascades in different cell types [45, 46]. Abnormal MAPK regulation may occur in several models of CNS inflammation and injury [19]. Previously, we have demonstrated that MAPKs such as ERK1/2 were essentially required for upregulation of MMP-9 by BK [14, 30]. Here, our data showed that activation of ERK1/2 participated in PMA-induced MMP-9 expression in RBA cells (Fig. 4), which was activated via Nox/ROS-dependent pathway. Moreover, rottlerin reduced MMP-9 expression by inhibiting PMA-stimulated ERK1/2 MAPK activation in RBA cells (Fig. 4). These results suggest that rottlerin can block PMA-induced MMP-9 expression through reducing PKC-δ-mediated activation of Nox/ROS-ERK1/2 cascade in RBA cells. The findings are similar with the report showed that PMA induces MUC16 expression via PKC-δ and p38 MAPK, but not ERK1/2, in human airway epithelial cells [47]. Moreover, another study indicated that the activation of PKC-δ induced cell growth arrest in NPA cells, through an ERK-dependent pathway. PKC-δ may be an effective molecular target for novel therapy in thyroid cancer [48]. In contrast with the previous study showed that rottlerin enhances IL-1β-induced COX-2 expression through sustained p38 MAPK activation in MDA-MB-231 human breast cancer cells [49], these differences suggest that the nature of its effects may vary in a stimuli-dependent or cell-type-specific manner.

The progressive increase of oxidative stress during injuries not only causes oxidative damage to cellular macromolecules, but also modulates the pattern of gene expression through functional alterations of transcription factors. The transcription factors such as AP-1 play a key role in the regulation of several gene expressions including MMP-9 during inflammation, cell proliferation, and apoptosis associated with physiological and pathological events [50]. In addition, several reports also indicate that AP-1 is involved in the pathogenesis of brain inflammation [20]. In the CNS, various stimuli can induce expression of several inflammatory mediators such as MMP-9 through ROS-mediated activation of AP-1 manner in astrocytes [20]. Previously, we have demonstrated that AP-1 participates in upregulation of several genes including MMP-9 by proinflammatory factors through ROS-dependent manner [30, 51]. These results implicate that AP-1 may play a critical role in upregulating MMP-9 expression and lead to inflammatory gene expression in pathological events including the CNS inflammation [20]. Therefore, we focus on the effects of rottlerin on PMA-stimulated activation of the transcription factor AP-1 in RBA cells. The results showed that PMA-induced MMP-9 expression is mediated through up-regulation of AP-1 (Fig. 5). The Nox/ROS and ERK1/2 were involved in PMA-stimulated c-Fos/AP-1 expression. Moreover, PMA-stimulated increase of AP-1 (c-Fos induction), MMP-9 promoter activity, and MMP-9 mRNA expression were significantly inhibited by rottlerin (Fig. 5b–d). These results suggested that rottlerin may alleviate upregulation of MMP-9 by PMA via inhibiting activation of the transcription factor AP-1 in brain astrocytes. Moreover, our data also showed that PMA directly induces MMP-9 expression via PKC-δ-mediated Nox/ROS and ERK1/2 signals, linking to activation of c-Fos/AP-1, which results in the brain astrocytic (RBA) migration (Fig. 6).

## Conclusions

**Fig. 7:**
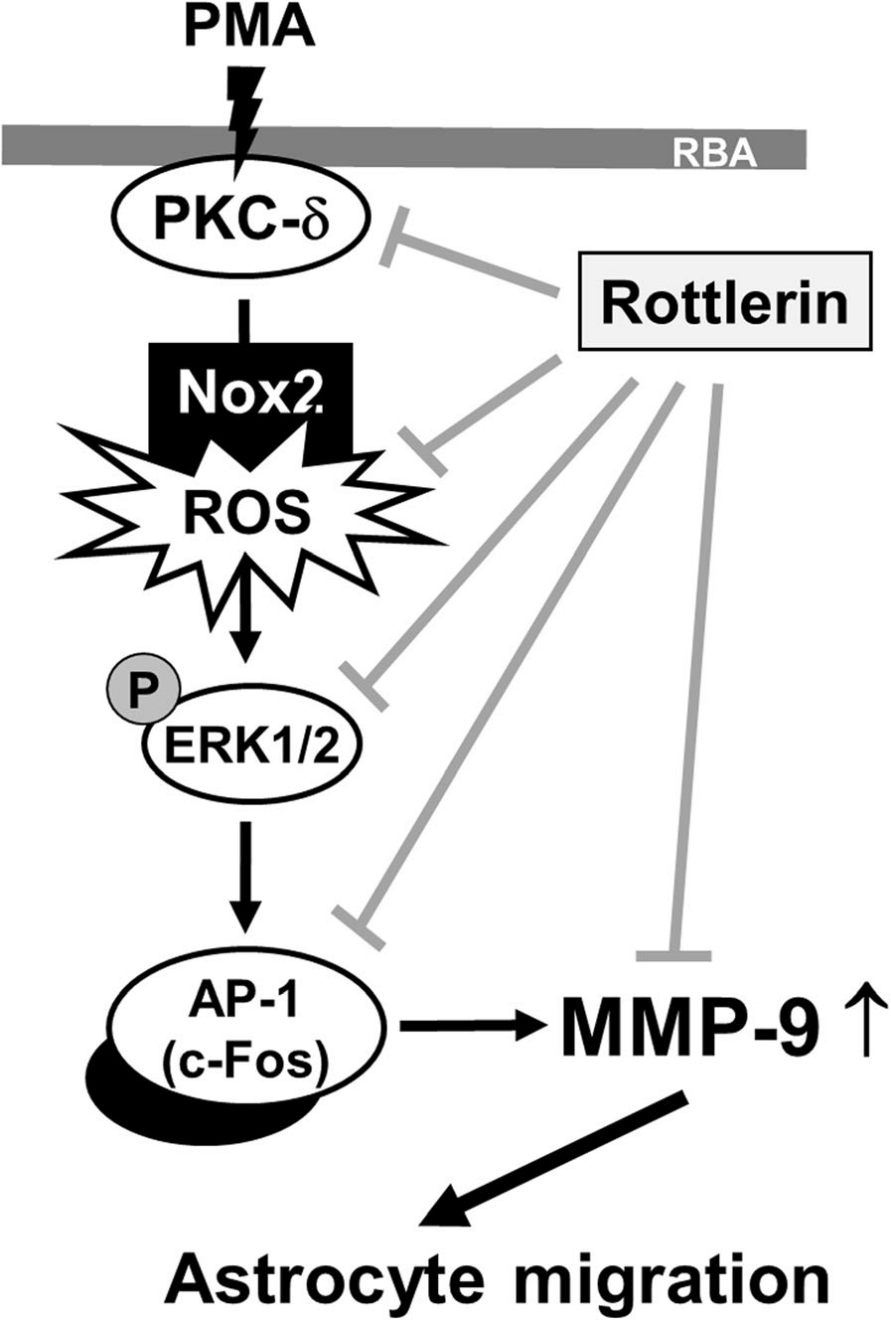
Schematic presentation of the effects of rottlerin on the PMA-induced MMP-9 expression and astrocytic migration. In brain astrocytes (RBA cells), PMA induces ERK1/2 activation through PKC-δ and Nox-derived ROS signals resulting in c-Fos/AP-1-dependent MMP-9 expression. The increased MMP-9 leads to RBA cell migration. Rottlerin suppressed the PMA-induced MMP-9-related events (brain astrocytic migration) via reducing activation of PKC-δ, Nox/ROS, ERK1/2, and c-Fos/AP-1 signaling pathways

Based on the observations from literatures and our findings, Fig. 7 depicts a model for the inhibitory action of rottlerin on PMA-induced MMP-9-dependent events like RBA cell migration. Herein, the data showed that pretreatment with rottlerin can inhibit activation of several signaling molecules in PMA-induced MMP-9 expression, including PKC-δ, Nox/ROS, ERK1/2, and c-Fos/AP-1. These findings concerning the natural product rottlerin-reduced PMA-induced PKC-δ-mediated Nox/ROS signal, MAPKs (i.e., ERK1/2), and MMP-9 expression in brain astrocytes imply that rottlerin (as a PKC-δ inhibitor) may play a critical role in the anti-oxidative and anti-inflammatory properties. Rottlerin may be as a neuroprotective natural product in several brain inflammatory disorders.

## Data Availability

The datasets used and/or analyzed during the current study are available from the corresponding author on reasonable request.

## Abbreviations

CNS: Central nervous system
AD: Alzheimer’s disease
ECM: Extracellular matrix
PMA: Phorbol 12-myristate 13-acetate
MMP-9: Matrix metalloproteinase-9
PKC: Protein kinase C
ROS: Reactive oxygen species
RBA: Rat brain astrocytes
ERK: Extracellular signal-regulated kinase
GAPDH: Glyceraldehyde-3-phosphate dehydrogenase
DMEM/F-12: Dulbecco’s modified Eagle’s medium/Ham’s nutrient mixture F-12
FBS: Fetal bovine serum
ECL: Enhanced chemiluminescence
BCA: Bicinchoninic acid
PBS: Phosphate-buffered saline
RT-PCR: Reverse transcription-polymerase chain reaction
DCF-DA: 2′,7′-Dichlorofluorescein diacetate
NAC: n-acetyl-cysteine
